# Bœnninghausen’s Aphorisms of Hippocrates

**Published:** 1887-06

**Authors:** 


### BŒNNINGHAUSEN'S APHORISMS OF HIPPOCRATES.

### Extracts Translated by Dr. A. McNeil, of San Francisco, Cal.

### Ileus.

We hope the kind reader will pardon us if we speak on this one occasion of ourself, and our never-to-be-forgotten teacher and friend, Hahnemann. It was toward the end of March, 1833, when we were attacked by this disease (ileus). The *right ileum* was the seat of the uncommonly painful suffering, which continued fourteen days. Four physicians, of whom our honored friend, Medical Counselor Dr. Aegidi, at that time Physician-in-ordinary to the Princess Friedrich, of Düsseldorf, only lives and can testify to this truth, hastened to our rescue and to counsel with each other, but in vain. We first, in the middle of the last fourteenth night, full of inexpressible torment, had the good fortune ourself to discover the remedy which had *hitherto never* been administered for this disease. This was Thuja, to which we were directed by the circumstance that only the *uncovered parts sweat*, and that profusely, *while the covered parts remained dry and hot*—a symptom which belongs only to Thuja, and is overlooked even by C. W. Wolf. A pellet of Thuja$^{30}$ brought relief of the pains in five minutes, and in ten a profuse movement of the bowels, followed immediately by a refreshing sleep, from which we awoke next morning as if newly born. We were taking a hearty breakfast, which was relished very much, when our four friends came into the room, full of joy and surprise, and still more astonished when they heard the remedy that had done it. We soon communicated the history of the case to our honored Hahnemann, but did not receive a reply till April 28th, 1833, because he had been suffering from a dangerous suffocative catarrh from April 3d to 24th, and was near to death. In this letter of reply, which is now before us, of six pages are the following, written as if by divination: "In giving you supplementary advice as to restoring the activity of your intestines, let me call your attention to Conium and Lycopodium, and to daily walks in the open air. It is delightful that you have done justice to the so beneficent remedy, Thuja, by your example." And how wonderful this advice agreed with that which had been literally followed before it had been received by us! We had, *two days* after sending our letter to Hahnemann, owing to the change of symptoms which had occurred, taken Conium, and the evening before the receiving of this letter taken Lycopodium. No other medicine was taken afterward, because every trace of the ileus had disappeared, and till this hour has not returned. This fact, it appears to us, proves even more than the most accurate knowledge of the course of a not frequent disease, and of the individual virtues of remedies; but it appears to justify the presumption of a peculiar inspiration, and truly such a one as enabled the acute founder of Homœopathy to perceive that such things as Gold, Table-salt, Sepia, Silicea, and Lycopodium were not only real medicines, but by his unequaled provings of them demonstrated that they were most efficacious and indispensable. We do not believe that the long history of medicine has a single fact to parallel this one, or to excel it in wonderfulness.

